# Pancreatic Stone Protein as an Emerging Biomarker in Paediatric Nosocomial and Postoperative Sepsis: An Integrative Review of Diagnostic and Prognostic Performance

**DOI:** 10.3390/ijms27114827

**Published:** 2026-05-27

**Authors:** Adrian Ramírez Quintana, Elena Laura Gaman, Mihaela Badea, Elena Mihaela Constantinescu

**Affiliations:** 1 Department of Fundamental, Prophylactic and Clinical Disciplines, Faculty of Medicine, Transilvania University of Brasov, 56 Nicolae Balcescu, 500019 Brasov, Romania; adrian.ramirez@unitbv.ro (A.R.Q.); elena.constantinescu@unitbv.ro (E.M.C.); 2 Faculty of Medicine, Carol Davila University of Medicine and Pharmacy, Bdul Eroilor Sanitari 8, 050474 Bucharest, Romania; gaman.laura@umfcd.ro; 3 Research Center for Fundamental Research and Prevention Strategies in Medicine, Research and Development Institute, Transilvania University of Brasov, 10 Institutului St., 500484 Brasov, Romania

**Keywords:** pancreatic stone protein, nosocomial infections, biomarker, postoperative infection, pediatric intensive care, systemic inflammatory response syndrome

## Abstract

Healthcare-associated infections remain a major cause of morbidity and mortality in paediatric patients, particularly among those undergoing surgery or requiring intensive care. Distinguishing postoperative sterile inflammation from early sepsis is clinically challenging because conventional biomarkers such as C-reactive protein (CRP) and procalcitonin lack sufficient specificity. This narrative review aimed to analyse current evidence on pancreatic stone protein (PSP) as an emerging biomarker for the detection and risk stratification of infection in paediatric populations. A structured literature search was performed in PubMed and Web of Science for studies published between 2020 and 2026 using terms related to nosocomial infections, paediatric surgery, and PSP, identifying 147 articles; 54 met the inclusion criteria after screening. The analysed studies show that PSP levels increase significantly in children with sepsis compared with those with non-infectious systemic inflammation and are associated with bacteraemia, organ dysfunction, and mortality. In several paediatric cohorts, PSP demonstrated diagnostic performance comparable to or better than traditional markers, with reported AUROC values up to approximately 0.82 for distinguishing sepsis from non-infectious inflammation. Evidence from neonatal and high-risk populations also suggests strong sensitivity and specificity for early infection detection. Overall, PSP appears to be a promising biomarker for the early identification and prognostic stratification of paediatric sepsis; however, variability among studies highlights the need for larger multicentre prospective investigations and for integrating PSP into multimarker diagnostic algorithms.

## 1. Introduction

Healthcare-associated infections in paediatrics are a significant cause of sepsis, admission to paediatric intensive care units (PICUs) and mortality, with a particular impact on patients undergoing major surgery, mechanical ventilation and prolonged invasive devices [1,2,3,4]. In paediatric postoperative care, the combination of surgical stress, systemic inflammatory response and stay in the PICU promotes the onset of nosocomial infections, including catheter-related bacteraemia and complicated abdominal foci, which can rapidly progress to sepsis and septic shock [1,3,4,5]. In this context, distinguishing between sterile inflammation associated with surgery and hospital-acquired sepsis is particularly complex and clinically critical.

Classic inflammatory markers, such as C-reactive protein (CRP) and procalcitonin (PCT), are routinely used to guide the diagnosis of infection in critically ill children; however, their specificity is limited because they also increase in non-infectious inflammatory states, such as surgical stress or the systemic response to tissue damage [1,2,6,7,8]. In paediatric sepsis, several studies have shown that, although CRP and PCT provide useful information, their discriminatory capacity to differentiate sepsis from non-infectious systemic inflammatory response syndrome (SIRS) is not optimal, with areas under the curve (AUC) often around 0.6–0.7 [1,2,6,8]. This limitation translates into broad empirical administration of antibiotics, risk of therapeutic delay in true sepsis, and contribution to antimicrobial resistance [4,7,9,10]. Hence the growing interest in more specific biomarkers that enable early infection detection and more accurate risk stratification.

In this scenario, Pancreatic Stone Protein (PSP) has been proposed as an emerging biomarker with high diagnostic and prognostic potential in sepsis. A recent meta-analysis evaluating the diagnostic accuracy of PSP across different populations with suspected sepsis reported an overall sensitivity of approximately 0.88 and a specificity of approximately 0.78, with an area under the summary receiver operating characteristic curve of approximately 0.90. In the sub-analysis of newborns, sensitivity reached around 0.91, with moderate specificity, supporting its usefulness in detecting sepsis, especially at an early age [11].

A previous systematic review focusing on infections in hospitalised adults showed that, using a cut-off point close to 44 ng/mL, PSP offered an AUC of 0.81 for the diagnosis of infection, outperforming CRP and PCT, and further improving its performance when combined with CRP [7]. Another narrative review summarised the available evidence and concluded that PSP tends to have better diagnostic performance in identifying infection than conventional biomarkers, and provides relevant prognostic information in sepsis [12,13].

In critically ill paediatric patients, several recent studies have specifically analysed PSP behaviour. In a retrospective cohort of 97 children admitted to the PICU with new-onset sepsis, PSP levels measured in the first 24 h after diagnosis were significantly higher in patients with positive blood cultures than in those with negative blood cultures, and increased progressively from non-septic patients to sepsis and septic shock. These findings suggest that PSP could be useful both for detecting bacteraemia and for stratifying the risk of progression to septic shock in paediatric PICUs [5].

In a prospective cohort study conducted in a single PICU and a paediatric high-dependency unit, which included 99 children with signs of SIRS, the diagnostic accuracy of PSP, PCT, and CRP for identifying sepsis was evaluated. The prevalence of sepsis was 35.4%, and PSP levels were significantly higher in patients with sepsis compared to those with non-infectious systemic inflammation. Thus, PSP appears to be a marker with better overall performance for discriminating sepsis from non-infectious SIRS in critically ill children [6].

A previous pilot study in 40 paediatric patients with sepsis or SIRS analysed PSP kinetics during the first week of evolution. In this study, children with sepsis had higher PSP concentrations than those with non-infectious inflammation from day 1, and these levels were associated with organ dysfunction scores and vital outcomes. These results reinforce the role of PSP not only as a diagnostic tool but also as an indicator of severity and prognosis in critically ill children [2].

In the neonatal setting, late-onset sepsis, often associated with prolonged hospitalisation and invasive devices, is an example of nosocomial infection in a highly vulnerable population. A case–control study involving 40 neonates with late-onset sepsis and 40 healthy controls found that serum PSP concentrations were significantly higher in both sepsis subgroups (with and without positive blood culture) compared to controls [14].

From a pathophysiological point of view, it has been suggested that PSP/reg is closely related to pancreatic injury in the context of sepsis. A study involving 137 children with sepsis admitted to the PICU showed that circulating PSP/reg levels were higher in patients with septic shock than in those with less severe sepsis, and that they increased significantly in cases with marked elevations in pancreatic amylase. Non-survivors had higher PSP/reg concentrations than survivors, suggesting an association between this biomarker, pancreatic involvement, and prognosis. These findings support the hypothesis that PSP/reg is actively involved in the inflammatory response and in protecting against pancreatic injury during sepsis, thereby reinforcing its role as a biomarker of organ dysfunction [15].

Although most paediatric studies focus on sepsis in general (community-acquired and nosocomial) and do not specifically analyse postoperative sepsis in children, evidence from adults undergoing complex abdominal surgery is useful for extrapolating concepts to the paediatric setting. Consistent with the widely reviewed concept that PSP tends to rise several days before the clinical manifestation of nosocomial sepsis in different hospital settings, its potential incorporation into screening strategies in high-risk surgical patients is supported [3].

Several reviews have also emphasised that PSP appears to increase earlier than other classic biomarkers and that its behaviour is less influenced by sterile inflammation, which is particularly relevant in the postoperative period, where surgical trauma can nonspecifically elevate markers such as CRP or PCT [12,13,16]. In hospitalised adults, meta-analyses of individual data have shown that PSP improves infection detection and helps to discriminate between infection and non-infection when combined with CRP, increasing the AUC to approximately 0.90 and improving both sensitivity and specificity compared to the isolated use of each marker [7,17]. In the emergency department, PSP has been significantly associated with the presence of sepsis, although its isolated performance has not been sufficient to recommend its use as the sole diagnostic marker, suggesting its integration into models combined with other clinical and analytical parameters [9].

Overall, the available evidence in the paediatric population suggests that PSP rises significantly in sepsis compared with non-infectious inflammation, discriminates between septic and non-septic conditions with good accuracy in PICUs and high-dependency units, and is associated with severity, organ dysfunction, and, in some studies, mortality [1,2,5,6,15,18]. In particularly vulnerable subgroups, such as newborns with late-onset sepsis or children with febrile neutropenia due to haematological malignancies, PSP has shown high sensitivity and specificity for detecting sepsis, even when blood cultures are negative [11,14,18]. In adults undergoing complex abdominal surgery, serial monitoring of PSP has provided valuable information for the early diagnosis of postoperative sepsis, opening up the possibility of extrapolating this approach to major paediatric surgery [3,4,13]. On this basis, it is proposed that the incorporation of PSP and other pancreatic biomarkers into specific diagnostic algorithms for the paediatric postoperative period could improve the early detection of nosocomial sepsis, facilitate a more rational use of antibiotics and, ultimately, contribute to reducing the morbidity and mortality associated with postoperative sepsis in children [1,2,3,4,5,6,7,9,10,11,12,13,14,15,16,17,18,19].

Despite the growing body of evidence on PSP in adult sepsis, three substantial gaps remain in the paediatric literature. First, most published paediatric studies are single-centre, retrospective, and have limited sample sizes, resulting in considerable variability in reported cut-off values and diagnostic performance metrics. Second, the molecular mechanisms underlying PSP elevation in paediatric sepsis—particularly those governing developmental differences between neonates, infants, and older children—have not been integrated into clinical interpretation, leaving cut-off selection largely empirical. Third, the translational obstacles preventing PSP from entering routine paediatric practice, despite more than a decade of clinical investigation, have not been systematically analysed.

The present narrative review aims to address these gaps by: (i) synthesising the current clinical evidence on PSP for the diagnosis and prognostic stratification of nosocomial sepsis in paediatric populations, with particular attention to postoperative, intensive care, neonatal, and oncohaematological settings; (ii) providing an integrated mechanistic framework that connects PSP’s molecular biology to its clinical behaviour and its variation across paediatric age groups; and (iii) critically analysing the practical and methodological barriers that currently limit its clinical translation. By integrating clinical, mechanistic, and translational perspectives within a single synthesis, this review seeks to provide a comprehensive reference for clinicians, researchers, and laboratory professionals working on paediatric sepsis biomarkers and to identify the priority directions for the next generation of clinical and translational studies.

## 2. Key Methodological Features and Diagnostic Performance of the Reviewed Studies

Nosocomial infections continue to be one of the most significant complications associated with paediatric hospitalisation, particularly in patients undergoing surgical procedures or with critical conditions requiring invasive interventions and prolonged hospital stays. A joint analysis of the included studies shows that the risk of infection in this group of patients is determined by a complex interaction among factors related to the host, medical interventions, and hospital microbiological ecology.

Several epidemiological studies have shown that paediatric patients undergoing major surgery have a significantly increased risk of developing hospital-acquired infections. In particular, patients with congenital heart disease undergoing cardiac surgery have a considerable incidence of postoperative infections, with a direct impact on the length of hospitalisation and morbidity associated with the surgical procedure [20]. Similarly, other studies in paediatric surgery have shown that perioperative metabolic alterations, such as dysglycaemia, can negatively influence postoperative recovery and increase the risk of infectious complications [21]. These findings suggest that the metabolic response to surgical stress is a relevant factor in susceptibility to nosocomial infections.

Table 1 summarises the methodological characteristics and diagnostic performance indicators of the studies directly evaluating PSP in paediatric or adult populations relevant to the present synthesis, together with selected comparator biomarker studies (CRP, PCT) that are explicitly compared with PSP in the text. Studies addressing the broader epidemiology of nosocomial infections, antimicrobial resistance, or infection-prevention interventions without PSP measurement are referenced in the narrative only where they directly motivate the diagnostic role of PSP and are not included in Table 1. In the neonatal context, immunological vulnerability and the need for invasive intensive support lead to a high incidence of hospital-acquired infections. Studies conducted in neonatal units have documented that immunological immaturity, prematurity, and prolonged exposure to invasive devices are determining factors in the development of nosocomial infections and associated mortality [22,23]. Large-scale epidemiological surveillance data confirm these observations and highlight the importance of continuous monitoring systems for identifying epidemiological trends and improving prevention strategies [24].

Another consistent finding in the literature analysed is the central role of invasive devices in the genesis of hospital-acquired infections. Central venous catheter-associated bacteraemia remains one of the most common infectious complications in hospitalised patients. Recent studies have shown that the implementation of strict catheter management protocols can significantly reduce the incidence of device-associated infections, especially in paediatric and neonatal units [30,31]. These results highlight the importance of prevention strategies based on infection control measures and hospital surveillance.

## 3. Pediatric Epidemiological Context: Factors Shaping the Need for a More Specific Biomarker

A detailed review of the epidemiological literature highlights three characteristics of pediatric nosocomial infections that justify the search for a biomarker that is more specific than CRP or PCT. Firstly, pediatric patients undergoing major surgery—particularly congenital heart surgery—and critically ill newborns are exposed to a considerable baseline risk of postoperative and device-associated infection, with reported incidence rates and outcomes varying widely depending on the setting [20,22,23,32]. Secondly, the growing prevalence of multidrug-resistant Gram-negative pathogens, including ESKAPEEc organisms and carbapenemase-producing Enterobacterales, limits empirical treatment options and increases the clinical cost of diagnostic uncertainty [33,34,35,36]. Thirdly, conventional clinical predictors of nosocomial infection (length of stay, prior antibiotic exposure, invasive devices, severity of underlying disease) tend to identify already established clinical situations rather than incipient infections, limiting their predictive value for early intervention [30,31,37].

Two emerging lines of evidence strengthen the case for early molecular biomarkers in this population. In pediatric burn injuries, it has been demonstrated that plasma elevations of HSP90, HMGB1, FAS(APO) and AKT/mTOR phosphoproteins within the first 72 h predict subsequent nosocomial infection, suggesting that the host’s immune status—and not merely exposure to the pathogen—determines individual susceptibility [38]. In parallel, perioperative metabolic abnormalities, such as dysglycaemia, have been associated with higher rates of surgical site infection and sepsis following non-cardiac pediatric surgery [21]. These observations support a paradigm in which early host-derived molecular signals—with PSP as a representative example—could complement conventional surveillance and guide a more rational use of antibiotics.

The main methodological limitations of this body of literature are well known and apply to the studies analyzed throughout this review: predominantly retrospective and single-centre designs, heterogeneous case definitions, and a limited exploration of host-side determinants influencing susceptibility to infection.

## 4. Pancreatic Stone Protein: Clinical Evidence in Paediatric Nosocomial Sepsis

Overall, the available evidence suggests that paediatric nosocomial infections result from a complex interaction among host factors, hospital exposure, and microbiological characteristics. Although numerous clinical risk factors have been identified, the ability to identify patients at higher risk of developing infection early remains limited. Therefore, future research should focus on prospective multicentre studies that integrate clinical, microbiological, and immunological variables to develop more accurate diagnostic tools and more effective prevention strategies.

Paediatric studies indicate that PSP is a useful biomarker, but its performance varies considerably across populations, clinical contexts, and comparators. In general paediatric intensive care units, Bottari reported that PSP discriminates between sepsis and non-infectious inflammation with an AUROC of 0.82 at a cut-off of 123 ng/mL, a value that represents moderate to good diagnostic accuracy by conventional criteria (excellent: AUROC ≥ 0.90) with specificity of 89%, marginally outperforming PCT and CRP in this single-centre cohort, although sensitivity was only 63%, which positions PSP as a confirmatory rather than rule-out test in critically ill children [6]. In an earlier pilot study by the same group, initial diagnostic performance was more modest (AUC 0.636), but PSP stood out as a predictor of mortality (AUC 0.814), with discriminative capacity similar to PCT and superior to CRP, suggesting that its greatest strength may lie in prognostic stratification rather than in the binary diagnosis of sepsis [2]. In the Turkish paediatric cohort reported by Dündar et al. [25], PSP was significantly higher in confirmed sepsis and in non-survivors, with high sensitivities for both diagnosis (95%) and mortality prediction (92%); however, the corresponding AUROC values fell within the moderate range (0.67 and 0.71, respectively), again pointing to a sensitive but only moderately accurate biomarker at the overall discriminatory level. In broader paediatric sepsis cohorts, Saleh et al. [1] reported that PSP, copeptin, and APOA5 functioned as useful acute-phase reactants, with an AUROC of 0.868 for PSP in the diagnosis of sepsis (sensitivity 80%), although in this study, copeptin and APOA5 marginally outperformed PSP (AUC ~0.96). For mortality prediction, PSP and copeptin discriminated better than APOA5 but with only moderate prognostic accuracy (AUC ~0.70). Taken together, these results indicate that PSP’s discriminative performance in paediatric sepsis falls predominantly in the moderate to good range across studies, and that its comparative advantage over PCT and CRP, while consistent in direction, is generally marginal in magnitude and derives entirely from single-centre cohorts of limited sample size. In neonates, the behaviour of the biomarker is particularly robust: Rass reports an AUC of 0.87 with sensitivity of 96.2% and specificity of 88.5% for early-onset sepsis at a very low cut-off (12.96 ng/mL), with a high NPV, reinforcing the usefulness of PSP as a tool to ‘rule out’ sepsis and potentially shorten hospitalisation and antibiotics [26]. Wu confirms that PSP remains elevated in infected patients at 24 and 72 h and that serial measurements improve the AUC (~0.82), supporting dynamic monitoring strategies rather than a single cut-off point [27]. In high-risk settings such as febrile neutropenia in oncology and haematology, PSP offers a robust combination of sensitivity (0.84) and specificity (0.82), outperforming MR-proADM and CRP in identifying sepsis from the first day of the febrile episode [18].

By analysing the relationship with severity rather than infection *per se*, Zůrek shows that PSP/reg does not differentiate well between SIRS and sepsis until organ dysfunction appears, but rises markedly in patients with PELOD ≥ 12, MODS and in those who die, which aligns it more with a marker of multiple organ failure than with a simple marker of infection [28]. This view is consistent with the findings of Bottari [2,6] and Dundar [25], in which PSP is closely associated with mortality and severity scores 246. In addition, Schlapbach’s group [39] studied of reference values documents an age-dependent increase (medians of 2.6 ng/mL in very preterm infants, 6.3 in term neonates, and 16.1 in older children), which introduces a critical element for interpreting the different cut-off points proposed in the literature: the same absolute threshold can mean very different things in a neonate versus an older child.

Overall, paediatric studies agree that PSP tends to perform as well as or better than PCT and clearly better than CRP in distinguishing sepsis from non-infectious inflammation [2,6,18,31]. However, the magnitude of the AUC and the sensitivity/specificity balance vary: in neonates and febrile neutropenia, the profile is high and balanced [18,26], while in general paediatric ICUs, either better specificity (Bottari) or better sensitivity (Dundar) is observed, but rarely both at their maximum [6,31]. This suggests that the added value of the PSP could be different depending on the scenario: a ‘rule-out’ tool and reduction of antibiotics in early neonatal sepsis [26], a component of risk algorithms in febrile neutropenia [18], and a marker of severity and prognosis in paediatric ICUs [2,6,28,31], rather than a single definitive test for sepsis in all contexts.

Taken together, the available paediatric PSP evidence is encouraging but methodologically constrained. Reported diagnostic performance metrics fall predominantly in the moderate-to-good range (AUROC ~0.74–0.87) rather than the excellent range; cut-off values vary by approximately an order of magnitude across studies (from ~13 ng/mL in neonatal early-onset sepsis to >120 ng/mL in mixed paediatric SIRS cohorts); assay platforms differ between centers; and no multicenter external validation has yet been completed in paediatric populations. No paediatric study has so far demonstrated that PSP measurement, alone or in combination with conventional biomarkers, modifies clinically meaningful patient outcomes such as mortality, antibiotic exposure, or length of hospital stay. These limitations imply that the current evidence supports PSP as a promising candidate biomarker warranting further investigation but does not yet justify its incorporation into routine clinical decision-making in paediatric care.

## 5. Molecular Biology and Pathophysiological Mechanisms of PSP in Sepsis

### 5.1. The PSP/Reg Family: Structural and Functional Overview

Pancreatic stone protein (PSP), also designated regenerating islet-derived protein 1-alpha (*REG1A* in humans), is a secretory glycoprotein encoded by the *REG1A* gene on chromosome 2p12. It is synthesised as a 166-amino acid preprotein containing a 22-residue N-terminal signal peptide that is cleaved during secretion, yielding a mature 144-amino acid protein that is further processed by trypsin to produce a 133-amino acid glycoform [40,41]. PSP belongs to the broader Reg family of C-type lectin-like proteins, comprising four subclasses (Reg I, II, III, and IV) involved in tissue regeneration, antimicrobial defence, and inflammatory regulation [41,42]. PSP was historically identified independently by several research groups under different names, including lithostathine, pancreatic thread protein, and regenerating protein 1 (*Reg1*), before molecular characterisation demonstrated that these proteins are products of the same gene [40]. Although PSP was originally associated with the inhibition of calcium carbonate precipitation in pancreatic juice, it is now recognised as a multifunctional secretory protein with a central role in the systemic response to infection and tissue injury [40,43].

### 5.2. PSP in Systemic Inflammatory Pathways: Cytokine-Driven Induction

Mechanistic studies have established that PSP expression is induced by pro-inflammatory cytokines, particularly interleukin-6 (IL-6), released during tissue injury and systemic inflammation. Sekikawa et al. demonstrated in gastric epithelial cells that *REG1A* is a direct transcriptional target of the IL-6/STAT3 signalling axis and mediates the anti-apoptotic effects of STAT3 activation [44], a finding consistent with subsequent observations in pancreatic models showing that peri-acinar macrophage infiltration and local IL-6 release stimulate *REG-1* expression in adjacent acinar cells [45]. Reding et al. extended these findings *in vivo* by showing in murine and human studies that the pancreas senses remote organ damage and systemic stress and responds by secreting PSP into the circulation, even in the absence of direct pancreatic injury—supporting the concept of the exocrine pancreas as an “acute-phase organ” with a 100-fold higher PSP tissue content than any other organ [43]. Together, these data place PSP downstream of the principal innate immune cascades activated during sepsis and provide a coherent biological rationale for its early elevation in the circulation following pathogen recognition.

### 5.3. Modulation of Neutrophil Activation

The pivotal mechanistic work by Keel et al. demonstrated in a cohort of 83 trauma patients that circulating PSP levels doubled following trauma and increased more than tenfold in patients who subsequently developed sepsis. *In vitro*, recombinant PSP was shown to bind directly to polymorphonuclear neutrophils (PMNs) and induce their activation, evidenced by downregulation of L-selectin (CD62L) and upregulation of the integrin CD11b [46]. This activation profile, characteristic of primed neutrophils with enhanced adhesive capacity, indicates that PSP is not merely a passive marker of systemic inflammation but an active mediator that promotes leukocyte recruitment to sites of infection. The capacity of PSP to engage neutrophils through C-type lectin-like binding aligns with the structural classification of the Reg family and provides a molecular basis for its association with the systemic inflammatory cascade observed in paediatric and adult sepsis [40,46].

### 5.4. Inhibition of Pyroptosis and Protection of Pancreatic Acinar Cells

Recent experimental evidence has expanded the mechanistic understanding of PSP’s protective role in sepsis-associated pancreatic injury. Using a murine cecal ligation and puncture model and LPS-stimulated acinar cell cultures, Liu et al. demonstrated that PSP/reg administration significantly attenuates pyroptosis of pancreatic acinar cells through downregulation of the NLRP3 inflammasome, reduction of caspase-1 p20 activation, and decreased cleavage of gasdermin D (GSDMD-N) [15]. In the same study, PSP/reg treatment reduced serum levels of LDH, TNF-α, and IL-6 and lowered histological injury scores, suggesting that the elevation of circulating PSP in septic patients reflects not only acinar cell stress but also an active homeostatic response aimed at limiting the amplification of the inflammatory cascade and preserving pancreatic integrity during systemic infection.

### 5.5. Molecular Basis of the Association with Organ Dysfunction and Multiple Organ Failure

The progression from sepsis to septic shock and multiple organ dysfunction syndrome (MODS) is paralleled by progressively higher circulating PSP concentrations, a relationship consistently documented in paediatric cohorts. Žůrek et al. reported that PSP/reg did not reliably distinguish SIRS from sepsis in early stages but rose markedly in patients meeting criteria for PELOD ≥ 12 and MODS, and in non-survivors, indicating that PSP elevation reflects cumulative organ injury rather than infection *per se* [25]. Bottari et al. reported median PSP values increasing from 72 ng/mL in non-septic patients to 238 ng/mL in sepsis and 375 ng/mL in septic shock, with PSP outperforming CRP and PCT in stratifying severity [5]. Mechanistically, the association between PSP and organ failure can be interpreted through three convergent processes documented in experimental models:

- Sustained acinar cell stress driven by sepsis-induced microcirculatory dysfunction and cytokine exposure [15,43].
- Systemic amplification of innate immune signalling via IL-6/STAT3-mediated PSP induction in extrapancreatic tissues [44,45].
- Direct modulation of neutrophil and macrophage function leading to leukocyte recruitment and collateral tissue injury in distant organs including lung, kidney, and liver [46,47].

### 5.6. Developmental Differences in PSP Expression: Neonates Versus Older Children

The pathophysiological interpretation of PSP must account for age-dependent differences in baseline expression and inflammatory responsiveness. Studies of human fetal pancreas have documented that *REG1* mRNA levels exhibit a marked developmental induction, with a 20-fold increase after 16 weeks of gestation, expressed exclusively in acinar cells [39,48]. This developmental programme continues postnatally: Schlapbach et al. demonstrated significantly lower baseline PSP concentrations in very preterm neonates (median 2.6 ng/mL) compared with term neonates (6.3 ng/mL) and older children (16.1 ng/mL), with a progressive postnatal increase observed during the first days of life [29]. These developmental differences likely reflect the maturation of pancreatic acinar tissue, postnatal completion of acinar cell self-duplication during the first weeks of life, and gradual acquisition of innate immune competence. Importantly, these baseline differences imply that identical absolute PSP values may carry different diagnostic and prognostic significance depending on age: cut-off values validated in older children cannot be directly transposed to neonates, where lower baseline expression makes even modest elevations clinically meaningful. This biological reality underlies the markedly lower diagnostic cut-offs reported in neonatal studies (e.g., 12.96 ng/mL in Rass et al. [26]) compared with paediatric ICU cohorts (e.g., 123 ng/mL in Bottari et al. [6]).

Taken together, the available mechanistic evidence supports PSP as a multifunctional acute-phase protein at the intersection of innate immunity, epithelial cell protection, and organ injury signalling. Its elevation during sepsis is not merely a passive marker of inflammation but reflects an active biological process involving IL-6/STAT3-dependent transcriptional induction in both pancreatic and extrapancreatic tissues, modulation of inflammasome activity and pyroptosis in acinar cells, and direct activation of circulating neutrophils through integrin-mediated adhesion. These mechanisms provide a coherent biological rationale for the clinical observations summarised in subsequent sections and underscore the need for age-stratified interpretation of PSP measurements in paediatric populations.

## 6. Barriers to Clinical Translation and Implementation

Despite the favourable diagnostic performance reported across paediatric studies, several practical and methodological barriers continue to limit the widespread clinical adoption of PSP as a routine biomarker in paediatric care.

### 6.1. Accessibility and Availability of Detection Methods

Most published studies have measured PSP using laboratory-based enzyme-linked immunosorbent assay (ELISA), which is accurate but requires several hours of processing time, dedicated equipment, and trained personnel—limitations incompatible with the rapid decision-making required in acute paediatric sepsis. The development of nanofluidic point-of-care (POC) testing platforms (notably the abioSCOPE system) has partially addressed this constraint by enabling quantitative PSP measurement at the bedside within minutes [2,6]. However, POC platforms are not yet widely available in most paediatric intensive care units, particularly in middle- and low-income settings, and their integration into routine clinical workflows remains limited.

### 6.2. Cost Considerations

The cost of PSP measurement—both per-test consumables and capital investment in dedicated POC equipment—exceeds that of routine biomarkers such as CRP and PCT. In healthcare systems with constrained resources, the incremental cost of PSP testing must be justified by demonstrable improvements in clinical outcomes, antibiotic stewardship, or length of stay. Formal cost-effectiveness analyses in paediatric populations are currently lacking and represent a priority for future investigation.

### 6.3. Inter-Laboratory Standardisation and Quality Control

A critical and frequently underappreciated limitation is the absence of standardised reference materials, harmonised measurement protocols, and external quality assurance schemes for PSP across different laboratories and assay platforms. The substantial heterogeneity in cut-off values reported in the literature—ranging from 12.96 ng/mL in neonatal early-onset sepsis [26] to 167 ng/mL in mixed paediatric SIRS cohorts [2]—reflects not only genuine biological variability (age, clinical context, infection severity) but also methodological differences between ELISA-based and nanofluidic POC measurements, and the absence of international calibration standards. Until such standardisation efforts are completed, comparison of PSP values between centres, between studies, and across patient populations must be undertaken with caution, and threshold values reported in one setting cannot be assumed to apply universally.

### 6.4. Regulatory and Clinical-Implementation Considerations

The regulatory status of PSP assays varies between jurisdictions, with full clinical approval (CE marking, FDA clearance) available for some platforms but not all. Furthermore, in contrast to CRP and PCT—both of which have been incorporated into paediatric clinical practice guidelines—PSP is not yet included in major paediatric sepsis guidelines (e.g., the Surviving Sepsis Campaign paediatric guidelines, the Society of Critical Care Medicine recommendations). The absence of guideline endorsement constitutes a substantial barrier to routine adoption, as clinicians and institutions are unlikely to incorporate a novel biomarker into protocols without formal recommendations from recognised scientific bodies.

### 6.5. Need for Paediatric-Specific Validation and Age-Stratified Reference Ranges

Most of the methodological framework for PSP measurement was originally developed in adult populations. The need for paediatric-specific assay validation, age-stratified reference intervals, and population-appropriate cut-off values—particularly across neonatal, infant, and adolescent subgroups—remains an unmet need that further constrains the immediate clinical translation of PSP in paediatric care.

### 6.6. Limitations of the Present Review

The methodological limitations of this work as a narrative review should be explicitly acknowledged. The selection of studies was not performed by two independent reviewers in duplicate, no formal protocol was pre-registered (e.g., in PROSPERO or OSF), and no quantitative risk-of-bias assessment or evidence-grading exercise was undertaken. As a consequence, the conclusions drawn from this synthesis should be interpreted as a qualitative integration of the available paediatric PSP literature rather than as a systematically derived appraisal of evidence quality. Definitive answers regarding the diagnostic and prognostic value of PSP in paediatric nosocomial sepsis will require formal systematic reviews and meta-analyses based on the growing body of primary evidence in this area.

## 7. Materials and Methods

### 7.1. Study Design

**Figure 1:**
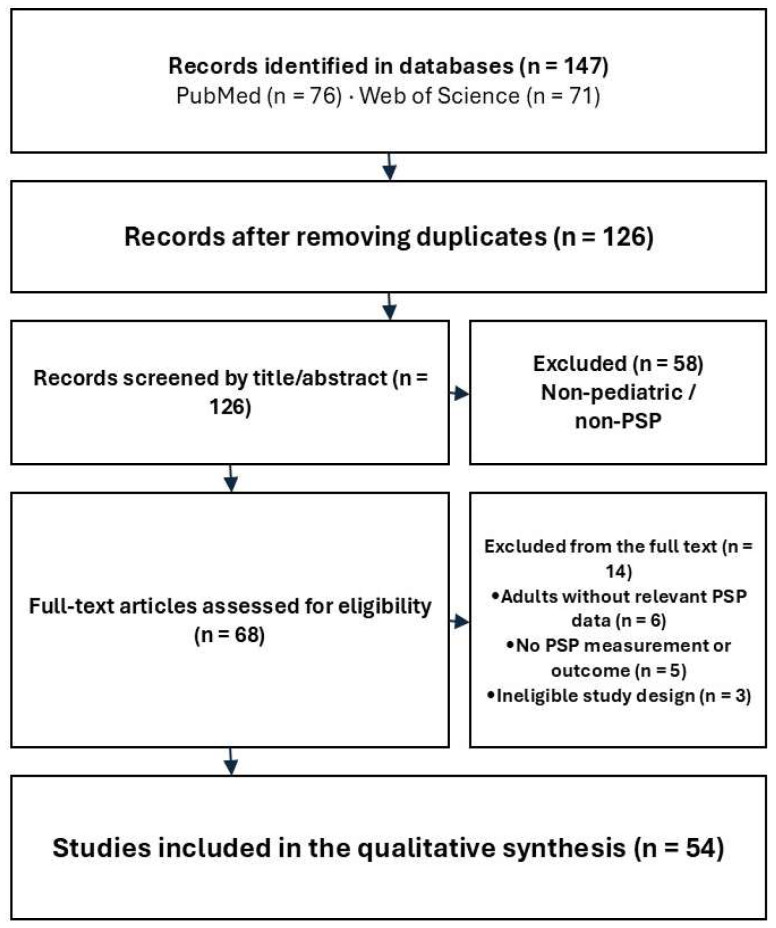
Research Prism Diagram.

This work is a structured narrative review. It does not constitute a systematic review or a scoping review and was not designed to meet the methodological standards of either format. In particular, the present synthesis does not include formal risk-of-bias assessment using validated instruments such as QUADAS-2 or the Newcastle–Ottawa Scale, nor does it apply evidence-grading methodologies such as GRADE. To enhance methodological transparency, however, several reporting elements inspired by the PRISMA 2020 statement and its Extension for Scoping Reviews (PRISMA-ScR) were adopted on a voluntary basis, including a documented database search strategy, pre-specified eligibility criteria, a recorded study selection process, and a flow diagram of inclusions and exclusions (Figure 1). These elements are intended to facilitate reproducibility and reader appraisal and should not be interpreted as evidence of systematic-review-level methodological rigour. A structured literature search was performed in PubMed/MEDLINE and Web of Science Core Collection covering the period from 1 January 2020 to 31 March 2026. The following search string was applied, with adaptation to the syntax of each database:

((“nosocomial infections” [MeSH] OR “nosocomial” OR “hospital-acquired”) AND (surgery OR surgical OR postoperative) AND (pediatric* OR paediatric* OR child* OR neonat*) AND (“Pancreatic Stone Protein” OR PSP OR “*Reg1*” OR biomarker OR sepsis)).

The reference lists of relevant articles were also screened manually to identify additional eligible studies.

### 7.2. Eligibility Criteria

Studies were considered eligible for inclusion if they fulfilled all of the following predefined criteria. Studies involving paediatric populations, defined as individuals aged 0–18 years according to the classifications established by the World Health Organisation (WHO) and the American Academy of Paediatrics, were considered. Eligible cohorts included neonatal (0–28 days), infant (1–12 months), childhood (1–12 years), and adolescent (13–18 years) subgroups. Investigations evaluating circulating pancreatic stone protein (PSP/*Reg1*) and/or related regenerating family biomarkers, including pancreatitis-associated protein (PAP/Reg3), quantified in serum or plasma specimens using validated analytical methodologies, were included for further analysis. Considering the outcomes, all the studies that met the criteria of reporting clinically relevant infectious and prognostic endpoints, including sepsis, healthcare-associated or nosocomial infections, postoperative infectious complications, organ dysfunction, multiple organ failure, or all-cause mortality, were considered. Study design was carefully analysed, including in the analysis the original peer-reviewed research articles encompassing prospective or retrospective cohort studies, case–control studies, cross-sectional diagnostic accuracy investigations, and randomised controlled trials. Ensuring inclusion of internationally accessible evidence while maintaining methodological consistency in data extraction and interpretation, publications available in English, Spanish, French, German, or Portuguese were included.

They were excluded from analysis studies conducted exclusively in adult populations (≥18 years), unless they provided direct mechanistic or comparative evidence relevant to PSP that could inform interpretation in paediatric populations; such adult studies were included exclusively as contextual references and clearly identified as such. Narrative reviews, systematic reviews, meta-analyses, editorials, letters, and conference abstracts, as the present work synthesises primary evidence; existing reviews were consulted to identify additional primary studies but were not included in the analytical synthesis. Non-indexed preprints, conference abstracts, and grey literature lacking formal peer-review evaluation were excluded to ensure methodological robustness, scientific validity, and reliability of the synthesized evidence. Studies were excluded if they did not report clinically relevant outcomes related to infection diagnosis, disease severity stratification, or prognosis, or if they were experimental animal or *in vitro* investigations without direct clinical applicability or translational relevance to paediatric patient care.

### 7.3. Study Selection Process

The search initially identified 76 records in PubMed and 71 in Web of Science, for a total of 147 articles. After removing 21 duplicate records, 126 unique articles underwent title and abstract screening. Of these, 58 were excluded for not addressing paediatric populations or for being unrelated to PSP, sepsis, or nosocomial infection. Sixty-eight full-text articles were then assessed for eligibility, and 14 were excluded for the following reasons: exclusively adult populations without PSP relevance (n = 6); absence of PSP measurement or relevant infection outcome (n = 5); and study design ineligibility (n = 3). A total of 54 articles were included in the final qualitative synthesis. The study selection process is summarised in Figure 1 (PRISMA-style flow diagram).

### 7.4. Data Extraction

For each included study, the following data were extracted: first author and year of publication, country, study design, population characteristics (age group, sample size), clinical setting, definition of infection or sepsis, biomarker(s) evaluated, measurement methodology, reported diagnostic performance indicators (sensitivity, specificity, AUROC, cut-off values), and main conclusions. The extracted data are presented in Table 1.

## 8. Conclusions

Pancreatic stone protein has emerged as a promising candidate biomarker in paediatric nosocomial sepsis, with consistent—though methodologically heterogeneous—evidence supporting its diagnostic value across neonatal, intensive care, and oncohaematological populations. Its principal clinical strengths suggested by the current evidence lie in three domains: a high negative predictive value for early infection detection in neonates, which supports its potential use as a rule-out tool to reduce unnecessary antibiotic exposure; discriminative performance equivalent to or marginally superior to CRP and PCT in distinguishing infectious sepsis from non-infectious systemic inflammation in paediatric intensive care; and progressive elevation with disease severity, supporting its role as an early indicator of organ dysfunction and mortality risk. Three core problems currently constrain the clinical translation of PSP in paediatric care. The first is the absence of standardised, age-stratified cut-off values, which prevents direct comparison between studies and limits the generalisability of reported diagnostic thresholds. The second is the methodological homogeneity of the existing evidence base—predominantly single-centre, retrospective, and small-sample—which has not yet been complemented by adequately powered multicentre prospective validation. The third is the absence of consensus on how PSP should be integrated into clinical decision-making: whether as a standalone test, as a component of multimarker algorithms, or as part of serial dynamic monitoring strategies.

Three priority directions should guide future research. First, large multicentre prospective studies are required, with standardised sepsis definitions, harmonised measurement methodologies, and age-stratified analyses across neonatal, infant, child, and adolescent subgroups. Second, the development and prospective validation of context-specific multimarker algorithms—combining PSP with PCT, CRP, lactate, and clinical severity scores—represents the most promising path to clinically actionable application. Third, formal cost-effectiveness analyses, clarification of the regulatory pathway, and incorporation of PSP into major paediatric sepsis guidelines are essential steps for translating the existing evidence base into routine clinical practice. In sum, PSP is not a universal solution to the diagnostic challenges of paediatric nosocomial sepsis, but it is a clinically valuable, biologically grounded biomarker whose place in paediatric care will depend on whether the research community can address the methodological, translational, and regulatory gaps that currently separate strong evidence from routine clinical use.

## Figures and Tables

**Table 1:** Methodological characteristics and diagnostic performance.

Study (Author, Year)	Population (Age)	Design (Country, Sample)	Definition of Infection/Event	Biomarker or Primary Factor	Key Results (Cut-Off/AUC/OR, etc.)	Main Conclusions
Bottari et al., 2024 [“High- Dependency Care”] [6]	Children > 1 month–<18 years with SIRS (with or without organ dysfunction)	Prospective cohort, single centre (Italy); 99 children in paediatric ICU and intermediate care	Sepsis: SIRS of infectious origin (clinical and/or microbiological criteria); non-sepsis: non-infectious systemic inflammation	PSP in venous blood, nanofluidic POC test, within the first 24 h of admission	Optimal PSP cut-off 123 ng/mL; AUROC 0.82 (95% CI 0.73–0.91); sensitivity 0.63 (0.43–0.80); specificity 0.89 (0.77–0.95). PCT AUROC 0.70; CRP AUROC 0.72	PSP was clearly more accurate than PCT and CRP in differentiating sepsis from non-infectious inflammation in critically ill children. A value ≥123 ng/mL increased the probability of sepsis (post-test probability ~73%), but the authors note that clinical uncertainty persists and recommend combining PSP with other biomarkers and clinical data.
Bottari et al., 2023 [pilot study] [2]	Children aged 1 month–18 years with sepsis or SIRS on admission to the emergency department/ICU/ward	Prospective pilot study (Italy); 40 critically ill paediatric patients	Sepsis vs. non-infectious SIRS according to clinical criteria and culture when available	PSP in ED on days 1, 2, 3, 5, and 7 from onset of signs of sepsis/SIRS (nanofluidic POC)	Day 1: cut-off 167 ng/mL; sensitivity 59% (CI 36–79%), specificity 83% (58–96%), AUC 0.636. For mortality: PSP AUC 0.814; PCT 0.814; CRP 0.657	PSP was higher in sepsis than in non-infectious inflammation and demonstrated good discriminatory capacity for survivors and non-survivors, similar to PCT and superior to CRP. Diagnostic utility for sepsis on day 1 was moderate, but the results support PSP as a promising biomarker requiring validation in larger cohorts.
Saleh et al., 2023 [1]	180 children with sepsis in paediatric ICU and 100 healthy controls	Prospective study (Egypt); single-centre paediatric ICU	Sepsis and subgroups (sepsis, severe sepsis, septic shock) according to clinical criteria; controls: healthy children	Serum PSP (along with copeptin and APOA5) was measured once within the first 24 h of admission	For sepsis diagnosis: PSP AUC 0.868; sensitivity 80%; (high specificity, not fully detailed in the abstract). For mortality: PSP AUC 0.709; sensitivity 74% e (moderate specificity). Copeptin and APOA5 AUC 0.960 and 0.965 for diagnosis	PSP, together with copeptin and APOA5, was significantly elevated in sepsis compared to controls. All were useful as acute phase reactants for diagnosing paediatric sepsis. PSP and copeptin discriminated between survivors and non-survivors, although their prognostic ability for mortality was only moderate; APOA5 was less powerful in predicting death
Dündar et al., 2024 [25]	Critically ill children in paediatric ICU with suspected or confirmed sepsis	Prospective cohort, single centre (Turkey); patients divided into confirmed sepsis (positive culture) vs. suspected sepsis, and survivors vs. non-survivors	Confirmed sepsis: positive blood culture; suspected sepsis: compatible clinical presentation without culture growth	Serum PSP, CRP, and PCT on admission; comparison with mortality rates	Sepsis diagnosis: PSP cut-off 50 ng/L, sensitivity 95%, AUC 0.67 (CI 0.52–0.81). Mortality prediction: sensitivity 92%, AUC 0.71 (0.56–0.83). PSP is more sensitive than CRP and PCT for confirming sepsis and predicting death	PSP was markedly elevated in confirmed sepsis and in non-survivors. It showed greater sensitivity than CRP and PCT both for confirming sepsis (with positive culture) and for predicting mortality, and correlated with other inflammatory markers and severity indices, supporting its integration into routine paediatric ICU practice.
Rass et al., 2016 [26]	Newborns with suspected early-onset sepsis (EOS), 52 infected and 52 uninfected	Prospective hospital study (Egypt); 104 neonates admitted to the NICU	EOS is defined by a compatible clinical presentation ± positive culture; subgroups: proven vs. probable infection	Serum PSP on admission; comparison with CRP	PSP cut-off 12.96 ng/mL: sensitivity 96.2%, specificity 88.5%, PPV 95.8%, NPV 89.3%, AUC 0.87. Significant positive e correlation between PSP and CRP in infected patients	PSP was clearly elevated in neonates with EOS compared to uninfected neonates. Its high sensitivity, specificity, and high negative predictive value indicate that it is a good marker for confirming and, above all, ruling out early neonatal sepsis, with the potential to reduce hospital stays and unnecessary antibiotic use.
Wu et al., 2017 [27]	119 neonates with suspected sepsis (0–7 days); 40 with “highly probable/probable” sepsis and 79 with possible or no sepsis	Prospective study (China); NICU, three- r sampling points	Neonatal sepsis is categorised as highly probable, probable, possible, or absent based on clinical and laboratory data	Serum PSP at 24, 72, and 168 h of life (ELISA)	Higher PSP in infected patients at all three time points. (*p* < 0.001). AUC ROC: 0.791 (CI 0.71–0.87) at 24 h and 0.790 (0.79–0.88) at 72 h; combination 24 + 72 h AUC 0.819 (0.74–0.90). Sensitivity ~0.84 and specificity ~0.82 for PSP (day 1) in distinguishing sepsis/non-sepsis	PSP was consistently superior in infected neonates and maintained good diagnostic performance in the first 72 h of life. The combination of serial values improved accuracy. It was concluded that PSP is a valuable biomarker for predicting neonatal infection, especially using repeated measurements.
Jiří et al., 2014 [28]	Children aged 0–19 years with SIRS or sepsis in the paediatric ICU	Prospective observational study over 5 days (Czech Republic); limited number of critically ill patients	SIRS and septic states are grouped; the onset of organ dysfunction, MODS, and mortality are recorded	PSP/reg measured serially while the patient met criteria for SIRS or sepsis	No cut-offs or AUCs reported; PSP/reg did not differentiate SIRS from sepsis until organ dysfunction appeared. Significantly higher levels in PELOD ≥ 12 or MODS; tendency for levels to be higher in deceased patients	PSP/reg was associated with severity, multiple organ failure, and death rather than with the mere presence of infection. The authors propose it as a marker of severity and risk of multiple organ failure in paediatric sepsis, rather than as a primary test for the diagnosis of sepsis.
Antari et al., 2023 [18]	70 episodes of febrile neutropenia in 70 children with leukaemia and lymphoma	Prospective cohort (Greece); follow-up to day 28	Sepsis/severe sepsis is defined by clinical criteria in the context of febrile neutropenia; 24% with documented bacterial/fungal infection.	PSP, MR-proADM, and CRP were measured on days 1, 3, and 7 of the febrile episode	Day 1: specificity PSP 0.82, MR-proADM 0.70, CRP 0.57; sensitivity PSP 0.84, MR-proADM 0.74, CRP 0.88. AUC for PSP ~0.80 for sepsis (higher than MR-proADM and CRP)	In children with cancer and febrile neutropenia, PSP and MR-proADM were promising biomarkers for the early diagnosis of sepsis, with PSP showing a better combination of sensitivity and specificity than CRP. PSP is suggested as a useful tool for stratifying sepsis risk in this high-risk group
Bottari et al., 2025 [5]	97 children admitted to paediatric ICU with new-onset sepsis (<24 h)	Retrospective cohort (Italy); comparison based on cultures and severity	Sepsis is classified by severity: non-septic, sepsis, septic shock; subgroups with and without bacteraemia	PSP within 24 h of diagnosis; comparison with CRP and PCT	Higher PSP with positive blood culture (median 108 vs. 82 ng/mL; *p* = 0.008) and with molecular methods (111 vs. 85 ng/mL; *p* = 0.026). Levels by severity: non-septic 72 ng/mL, sepsis 238 ng/mL, shock 375 ng/mL (*p* = 0.001). AUC PSP for predicting severity 0.75 (CI 0.64–0.87), superior to CRP (0.54) and PCT (0.60)	PSP reflected both the presence of bacteraemia and progression from sepsis to septic shock. Its performance in stratifying severity clearly exceeded that of CRP and PCT, supporting its use as a biomarker of severity in paediatric sepsis rather than just as a dichotomous e test for sepsis.
Schlapbach et al., 2015—reference values [29]	Newborns (very preterm and term), infants, and healthy children up to 16 years of age	Population-based cross-sectional study (Switzerland/Australia); 372 healthy subjects (217 neonates, 94 children, 61 adults)	Not applicable (subjects without infection; objective: to establish normal ranges)	Serum PSP by ELISA in different age groups and perinatal stages	Overall range 1.0–99.4 ng/mL; median 2.6 ng/mL in very preterm infants, 6.3 ng/mL in term newborns, 16.1 ng/mL in older children (*p* < 0.001). Higher PSP on day 3 postnatal than at birth	Age-specific normal PSP values were defined, with values increasing from birth to infancy. These ranges are useful for interpreting diagnostic cut-offs in neonatal and paediatric h y studies and for designing future clinical trials with PSP.

## Data Availability

No new data were created or analyzed in this study. Data sharing is not applicable to this article.
